# Pain and impact of pain on function in JIA-patients aged 10-19 years

**DOI:** 10.1186/1546-0096-9-S1-P157

**Published:** 2011-09-14

**Authors:** M Lanzinger

**Affiliations:** 1 Children and Adolescent Hospital, Skåne University Hospital, Lund, Sweden

## Background

Pain is a clinically significant symptom in children with JIA. The patients at our outpatient clinic rarely spontaneously report pain as an obstacle for functional ability.

## Objectives

To systematically screen for pain and functional disability due to pain in patients with JIA aged10-19.

## Methods

All patients aged 10-19, visiting the Paediatric Rheumatology outpatient clinic at Skane University Hospital, Lund, Sweden, in February to April 2010 were asked about presence of pain and how pain affected their functional capability.

Pain was measured with Visual Analogue Scale (VAS), 0-100 mm.

Impact of pain on functional capability in daily life was measured with Functional Disability Inventory (FDI). FDI consists of fifteen questions and a score is calculated where 0 indicates no, and 60 maximum impact on functional capability.

## Results

A total of 139 patients took part in the survey. Seventy-five of those had JIA, 45 girls and 30 boys. The results from this JIA-group are reported in this abstract.

The mean age of the patients was 15 years (range 10-19). Median pain measured on a VAS was 7 mm (range 0 to 84). 65% of the patients estimated pain between 0 and 25 mm on VAS. 23 patients (31%) reported no pain (VAS=0).

The mean value for FDI was 3 (range 0-22).

The two items in the FDI, which were scored highest, were participation in gymnastic lessons and to run 100 meters.

## Conclusions

A majority of the patients with JIA reports a low grade of pain, less than 25 mm on VAS 0-100 mm. Thirty-one percent of patients report no pain at all. This indicates a lower prevalence of pain as compared to other reports.

Taking part in physical activities seems to bee the area which is most affected by pain, according to FDI. Further studies should focus on factors that might influence the perception of pain in JIA such as subtype of disease, disease duration, age or gender.

